# Photobiomodulation Therapy Ameliorates Glutamatergic Dysfunction in Mice with Chronic Unpredictable Mild Stress-Induced Depression

**DOI:** 10.1155/2021/6678276

**Published:** 2021-03-29

**Authors:** Di Zhang, Qi Shen, Xiaolei Wu, Da Xing

**Affiliations:** ^1^MOE Key Laboratory of Laser Life Science & Institute of Laser Life Science, College of Biophotonics, South China Normal University, Guangzhou 510631, China; ^2^Guangdong Province Key Laboratory of Laser Life Science, College of Biophotonics, South China Normal University, Guangzhou 510631, China

## Abstract

Accumulating evidence indicates that dysfunction of the glutamatergic neurotransmission has been widely involved in the pathophysiology and treatment of depression. Photobiomodulation therapy (PBMT) has been demonstrated to regulate neuronal function both *in vitro* and *in vivo*. Herein, we aim to investigate whether the antidepressant phenotype of PBMT is associated with the improvement of glutamatergic dysfunction and to explore the mechanisms involved. Results showed that PBMT decreased extracellular glutamate levels via upregulation of glutamate transporter-1 (GLT-1) and rescued astrocyte loss in the cerebral cortex and hippocampus, which also alleviated dendritic atrophy and upregulated the expression of AMPA receptors on the postsynaptic membrane, ultimately exhibiting behaviorally significant antidepressant effects in mice exposed to chronic unpredictable mild stress (CUMS). Notably, PBMT also obtained similar antidepressant effects in a depressive mouse model subcutaneously injected with corticosterone (CORT). Evidence from *in vitro* mechanistic experiments demonstrated that PBMT treatment significantly increased both the GLT-1 mRNA and protein levels via the Akt/NF-*κ*B signaling pathway. NF-*κ*B-regulated transcription was in an Akt-dependent manner, while inhibition of Akt attenuated the DNA-binding efficiency of NF-*κ*B to the GLT-1 promoter. Importantly, *in vitro*, we further found that PKA activation was responsible for phosphorylation and surface levels of AMPA receptors induced by PBMT, which is likely to rescue excitatory synaptic transmission. Taken together, our research suggests that PBMT as a feasible therapeutic approach has great potential value to control the progression of depression.

## 1. Introduction

Depression is a severe mood disorder that affects more than 350 million people worldwide, causing a very large socioeconomic impact [1, 2]. However, the pathogenesis of depression is poorly known. Currently, medications available for treating depression are based on the dysfunction of the noradrenergic neurotransmission hypothesis, drugs that increase levels of brain norepinephrine (NE) or serotonin (5-HT) exert antidepressant effects [3, 4]. But the main limitation of current medications for depression is that drugs such as 5-HT selective reuptake inhibitor (SSRI) only make one-third of patients get remission, and drugs usually take several weeks or months to produce limited therapeutic responses [5, 6]. These research studies suggest that there may be other mechanisms involved in the pathogenesis of depression besides monoaminergic deficit that require further investigation.

It has been found that chronic stress continuously stimulates the hypothalamic-pituitary-adrenal (HPA) axis to induce the excessive release of glucocorticoids (corticosterone in rodents), which act on the corresponding glucocorticoid receptors, and then causes the glutamatergic neurons to release a large amount of glutamate [7–9]. It is known that increased glutamate causes neurotoxicity because excessive neurotransmitter release activates the influx of Ca^2+^ and increases efflux of K^+^, ultimately depolarizing the mitochondrial membrane, producing ROS, and oxidizing mitochondrial DNA [10, 11].

Perisynaptic astrocytes play a major role in maintaining the normal circulation and clearance of glutamate [12]. Glutamate transporter-1 (GLT-1, also known as excitatory amino acid transporter 2 (EAAT2) in humans) is the main glutamate transporter, which is mostly expressed by astrocytes and responsible for the reuptake of over 90% of glutamate [13]. Autopsy studies of brain samples from major depressive disorder (MDD) patients show a reduced density and a changed morphology of glial cells [14–19] and a decreased expression of astrocytic markers such as glial fibrillary acidic protein (GFAP) [20–23], excitatory amino acid transporters (EAATs), and glutamine synthetase (GS) in several brain regions [20, 24], particularly in the prefrontal cortex (PFC) and hippocampus. *In vivo* studies also provide evidence that pathological changes of astrocytes are related to the occurrence of depression because the density and numbers of astrocytes in the adult hippocampus and PFC of animals exposed to chronic stress are reduced [25–27].

Glutamate binding to *α*-amino-3-hydroxy-5-methyl-4-isoxazolepropionic acid (AMPA) receptors leads to postsynaptic membrane depolarization and mediates the rapid excitatory synaptic transmission of the central nervous system (CNS), which is closely related to the remodeling of neurons and the formation of synaptic plasticity [28, 29]. Several studies have shown that repeated restraint stress or chronic unpredictable stress can lead to the reduction of AMPA receptors on the postsynaptic membrane of rat prefrontal cortical neurons, inhibiting glutamatergic transmission [7, 30]. GluA1 is an important subunit of AMPA receptors; under the stimulation of N-methyl-D-aspartate (NMDA) receptors and activation of calcium/calmodulin-dependent protein kinase II (CaMKII), GluA1 drives AMPA receptors into synapses and ultimately leads to synaptic potentiation [31]. Phosphorylation of serine 831 and serine 845 in the GluA1 subunit regulates synaptic trafficking of GluA1-containing AMPA receptors during hippocampal long-term potentiation [32]. Additionally, the results suggested that chronic stress can reduce the expression of GluA1 subunits, decrease glutamatergic neurotransmission function, and then affect synaptic plasticity [33].

Changes in lighting conditions have a wide range of effects on physiological and behavioral functions, including circadian rhythm, mood, and cognition [34–36]. Irregular light environments can cause problems with circadian rhythms and sleep, ultimately leading to mood and learning impairments. In addition, a recent study has found that irregular light can also directly affect mood and learning without major disturbances to circadian rhythms and sleep. A growing body of research studies supports antidepressant effects of light therapy, while light deprivation can induce depressive-like behaviors, which further indicates that light signals are a powerful modulator of mood-related behaviors [37–43]. Studies have shown that light therapy can produce antidepressant effects by increasing serotonin levels [44]. As a novel and noninvasive therapy based on irradiating tissues with photons in the range of red to near-infrared (NIR) spectra (600-1100 nm), photobiomodulation therapy (PBMT) has shown the role of regulating neuronal functions in cell cultures, animal models, and clinical conditions [45, 46]. PBMT can efficiently penetrate into biological tissues including the CNS and produce beneficial photobiomodulation effects such as increasing ATP synthesis and stimulating neurogenesis [47]. There is evidence suggesting that cytochrome c oxidase (CcO) is the major mitochondrial chromophore of photobiomodulation, which has been considered the crucial photoacceptor of light in the range of red to NIR spectra [48–50]. Recently, several animal and clinical studies on MDD have shown that PBMT penetrating the PFC can induce antidepressant-like effects [51, 52]. However, the mechanism by which PBMT ameliorates glutamatergic dysfunction to display the antidepressant phenotype is unclear.

In this paper, chronic unpredictable mild stress (CUMS) and a depressive mouse model subcutaneously injected with corticosterone (CORT) were used to prepare animal models of depression, and the *in vivo* effects of PBMT on behavioral terms, glutamate levels, glutamate transporters, and glutamate receptors were detected in a variety of ways. The protective mechanism of PBMT was further studied by stimulating primary astrocytes and neurons with CORT to construct a pathological model *in vitro*. Overall, cellular and molecular studies have shown that PBMT can ameliorate glutamatergic dysfunction in depressive-like mouse models by improving glutamate uptake mediated by GLT-1 and glutamate receptor activity, ultimately achieving the effects of reducing glutamate excitotoxicity, increasing cell activity, and improving depression-like behaviors. Mechanically, photoactivation of transcription factor NF-*κ*B increased both the GLT-1 mRNA and protein levels, but inhibiting NF-*κ*B blocked the effects of PBMT. Furthermore, the transcription regulated by NF-*κ*B was Akt-dependent, while inhibition of Akt attenuated the DNA-binding efficiency between the NF-*κ*B and the GLT-1 promoter. Importantly, we further found that PKA activation was responsible for phosphorylation levels of S845 and surface levels of AMPA receptors induced by PBMT, suggesting that it is likely to rescue excitatory synaptic transmission. Thus, our research indicates that PBMT as a feasible treatment has great potential to control the progression of depression by targeting glutamate.

## 2. Materials and Methods

### 2.1. Materials

Corticosterone (CORT), rabbit monoclonal anti-phospho-AMPA receptor (pSer845), H89, 4′, 6-diamidino-2-phenylindole (DAPI), and poly-L-lysine solution were purchased from Sigma Chemical Corp. (St. Louis, MO, USA). The Mouse Glu ELISA Kit (SU-B20321) was purchased from Mlbio (Shanghai, China). RNAiso and Maxima Reverse Transcriptase were purchased from Takara (Takara, Inc., Osaka, Japan). The CCK-8 Kit was purchased from Dojindo (Kumamoto, Japan). API-2, PDTC, and KN93 were purchased from MCE (MedChemExpress, USA). DMEM, DMEM/F12 (1 : 1) medium, Neurobasal medium, and B27 supplements were purchased from Gibco Inc. (NY, USA). Mouse monoclonal GFAP (3670), rabbit polyclonal p-Akt (4060), mouse monoclonal p65 (6956), and rabbit polyclonal Na, K-ATPase (3010) were purchased from Cell Signaling Technology Inc. (BSN, USA). Rabbit polyclonal EAAT2/GLT-1 (ab41621), rabbit monoclonal Akt (ab179463), rabbit polyclonal p-PKA*α*/*β*/*γ* (ab75991), rabbit polyclonal AMPA receptor (ab183797), Alexa Fluor® 680 (ab175774), Alexa Fluor® 790 (ab175781), and Alexa 488/555-conjugated goat anti-mouse/rabbit anti-IgG (ab150113, ab150078) were purchased from Abcam Plc. (Cambridge, UK). Mouse monoclonal *β*-actin (sc47778), mouse monoclonal PKA*α*/*β*/*γ* (sc365615), and mouse monoclonal GAPDH (sc47724) were bought from Santa Cruz Bio. (CA, USA). Rabbit polyclonal MAP2 (17490-1-AP), rabbit polyclonal NeuN (26975-1-AP), and rabbit polyclonal PSD95 (20665-1-AP) were purchased from ProteinTech.

### 2.2. Animals

Five-week-old C57BL/6J male mice were purchased from Guangdong Medical Laboratory Animal Center, Guangzhou, China. Mice were housed in a standard laboratory environment (22 ± 2°C; 12 h light/dark cycle, with lights on from 07:00 to 19:00; and humidity: 50%-60%) and had free access to food and water except when mice were subjected to light/dark cycle disturbance or food/water deprivation stressors during the chronic unpredictable mild stress (CUMS) procedure. All of the experimental procedures were carried out in accordance with the protocols set and approved by the Institutional Animal Care and Use Committee of South China Normal University.

### 2.3. Experimental Design

#### 2.3.1. Experiment 1: CUMS Procedure and PBMT Treatment (Related to Figure 1(a))

C57BL/6J male mice were randomly divided into control and CUMS groups. The CUMS procedure was performed as previously described [53, 54], with some modifications, and the method's description partly reproduces their wording. CUMS mice were housed in individual cages and were exposed to a variable sequence of 10 unpredictable and mild stressors: (1) food deprivation for 24 h, (2) water deprivation for 24 h, (3) clip the tail for 1 min (clamp 1 cm from the tip of the tail), (4) cold swimming at 8°C for 10 min, (5) warm water swimming at 40°C for 10 min, (6) soiled bedding overnight (150 mL of water in 100 g of sawdust bedding per cage), (7) reversal of the light/dark cycle, (8) restriction for 2 h (mice were restrained in a 50 mL centrifuge tube with holes for 2 h), (9) cage tilting 45° overnight, and (10) odor overnight (50 g of SD rats' dirty sawdust bedding in 100 g of sawdust bedding per cage). Two kinds of stimulation were given at random every day, and the same kind of stimulation would not appear continuously to prevent habituation.

After 6 weeks of stimulation, the mice underwent the depression-related behavioral tests. And then, the CUMS group mice were randomly divided into two groups: CUMS and CUMS+PBMT groups. PBMT treatment was carried out for 30 days. During the PBMT treatment, these two groups of mice were still stimulated to avoid natural recovery. Behavioral tests were performed after PBMT treatment. All mice were sacrificed after behavior tests for the following experiments.

#### 2.3.2. Experiment 2: Corticosterone- (CORT-) Induced Mouse Model and PBMT Treatment (Related to Figure 1(h))

C57BL/6J male mice were injected with saline on three consecutive days before the start of the study to acclimate the mice to the injection procedure. Then, mice were randomly divided into control and CORT groups. The CORT group mice were administrated subcutaneously with CORT (Sigma-Aldrich, MO, USA) dissolved in 0.9% (*w*/*v*) saline containing 1% Tween-80 at 20 mg/kg. The control group mice were subcutaneously injected with an equal volume of the vehicle to mimic injection stress. CORT injection was administered between 09:00 and 09:30 a.m. daily for 28 days. Then, the mice underwent the depression-related behavioral tests. And then, the CORT group mice were randomly divided into two groups: CORT and CORT+PBMT groups. PBMT treatment was carried out for 30 days. During the PBMT treatment, mice were still injected to avoid natural recovery. Behavioral tests were performed after PBMT treatment. All mice were sacrificed after behavior tests for the following experiments.

Before constructing a mouse model of depression, a sucrose preference test was conducted, and only animals displaying an initial sucrose preference of more than 70% were selected for further experiments.

### 2.4. PBMT Treatment *In Vivo* and *In Vitro*

Our previous studies have shown that a laser with a dose of 2 J/cm^2^ is particularly effective in improving neuronal functions [50, 55–57]. In animal experiments, in order to receive the same dose of the laser in the hippocampus and cortex, the dynamometer was used to measure and calculate the actual power of the laser penetrating the skin and skull into the hippocampus. The penetration of PBMT through brain tissues is determined by the energy and attenuation coefficient [58]. After depilation of the head of the mice in the PBMT group (without removing the scalp and skull), they were all placed in the mouse fixture and only the head and tail were exposed, and the fiber was placed above the head of mice, while the tail did not receive the semiconductor laser (635 nm, NL-FBA-2.0-635, Light Photonics Corporation, Vancouver, WA; Laser Technology Application Research Institute, Guangzhou, China). Our previous study has found that the transmission of the laser through the skin and skull to the interior of the hippocampus was about 30%, which indicated that 6 J/cm^2^ PBMT irradiation is needed above the brain [50]. Mice were irradiated continuously by the laser for 10 min each day for 30 days. We measured that the dose reaching the interior of the hippocampus was 2 J/cm^2^, keeping the ambient temperature at 22 ± 1°C during PBMT and without increasing the local temperature of the head. The control, CUMS, and CORT groups of mice were kept in the same fixator for the same time as the PBMT group mice, but the laser source was not turned on (sham irradiation). The parameters of irradiation on mice are shown in Table S1.

PBMT treatment with cells was conducted as described in our previous study [55]. In short, cells were irradiated with the 635 nm laser for 1.25, 2.5, and 5 min in the dark, and the corresponding fluences were 1, 2, and 4 J/cm^2^, respectively. To minimize ambient light interference, when irradiated with the 635 nm laser, the cells are kept in a completely dark or an extremely dim environment. The parameters of irradiation on cells are shown in Table S2.

### 2.5. Behavioral Tests

#### 2.5.1. Sucrose Preference Test (SPT)

Anhedonia (reduced responsiveness to pleasurable stimuli) is a core symptom of depression and a phenotype that can be measured objectively in rodents. Sucrose preference was measured as previously described [59–61]. During the test, all mice were housed individually. Before the formal test, we trained mice to adapt to a 1% sucrose solution (*w*/*v*) for 48 h, during which two bottles of 1% sucrose solution were placed in each cage. Then, mice were deprived of food and water for 12 h. After deprivation, each cage was simultaneously provided with two weighed bottles, one bottle was 1% sucrose water, and the other one was pure water. After 1 h, two bottles were weighed again to calculate sucrose consumption. Sucrose preference was calculated according to the following formula: sucrose preferences (%) = sucrose intake/(sucrose intake + water intake) × 100%.

#### 2.5.2. Forced Swimming Test (FST)

The FST was initially used to screen antidepressant drugs [62]. The FST was conducted in an open cylindrical glass container 10 cm in diameter and 30 cm high filled with tap water up to 20 cm (25 ± 2°C) so that mice could not climb over the ridges. The FST was a two-day procedure, a 15 min trial was given on the first day for training, final tests were conducted for 6 min on the next day, and the durations of immobility were recorded by a camera during the last 4 min of the 6 min test and analyzed by Shanghai XinRuan software. Water was replaced in the glass container after each test. Immobility was defined as the minimal movement of the mice's tail and four limbs.

#### 2.5.3. Tail Suspension Test (TST)

The TST evaluates depression-like behavior based on the following observation: mice develop an immobile posture when suspended by their tails. Mice were hung 20 cm above the ground by medical tape placed about 1 cm from the tip of the tail. The TST continued for 5 min, and the durations of immobility were recorded by a camera during the last 4 min of the test. Immobile time was recorded and analyzed by Shanghai XinRuan software.

### 2.6. ELISA for Glutamate Detection

Glutamate was assessed using the Mouse Glu ELISA Kit (Mlbio, Shanghai). Brain tissues (cerebral cortex and hippocampus) were ground into a single cell suspension without cell lysis. The supernatant collected from brain tissues under the same conditions was used for the assay and was measured following the manufacturer's instructions. See the supplemental data for LC-MS analysis results.

### 2.7. Cell Culture and CORT Exposures

Cultures of primary neurons were performed as previously described [57, 63]. The cerebral cortex and hippocampus of neonatal C57BL/6J mice (1-2 days old) were dissected, digested with 0.25% trypsin, and mechanically dissociated. Isolated cells were resuspended in complete DMEM containing 10% heat-inactivated fetal bovine serum (FBS). After 6 h and every 2 days thereafter, the medium was replaced by the Neurobasal medium supplemented with 2% B27 (Invitrogen, USA), 2 mM L-glutamine, 100 U/mL penicillin, and 100 *μ*g/mL streptomycin (Gibco) and maintained at 37°C with 5% CO_2_.

Cultures of primary astrocytes were performed as previously described [63]. Astrocyte cultures were prepared from the cerebral cortex and hippocampus of neonatal mice (1-2 days old) and cultured in the astroglial medium (Dulbecco's modified Eagle's medium/Nutrient Mixture F-12 (DMEM/F12)) (1 : 1) (Gibco) containing 10% heat-inactivated FBS (*v*/*v*), 100 U/mL penicillin, and 100 *μ*g/mL streptomycin (Gibco). Cells were plated in 6-well plates at a uniform density of 30,000 cells/cm^2^ and were maintained in a 5% CO_2_ incubator at 37°C. At 9-11 days in culture, astrocyte cultures reached confluency, the cells were shaken at 260 rpm at 37°C for 8 h to remove microglia and oligodendrocytes, and the detached cells were subsequently removed. The cultures were treated with 0.25% trypsin/0.02% EDTA, and the disassociated cells were replated in 6- or 12-well plates. The plates and glass coverslips were previously coated with poly-L-lysine (100 *μ*g/mL). The medium was replaced every 2 days until the cells were confluent. At that time, the culture consisted mostly of astrocytes.

CORT was dissolved initially in culture-grade DMSO and then in culture media (final concentration of DMSO < 0.1%). Eight-day primary neurons and the third-generation primary astrocytes were used in all experiments in this study for CORT exposure. The cells were seeded in 6- or 96-well plates and cotreated with CORT for 24 h. And the control group was treated with the same dose of DMSO, which did not affect cell viability.

### 2.8. Cell Viability Assay

The cell viability was determined by using the Cell Counting Kit-8 (Dojindo, Kumamoto, Japan) assay. Briefly, primary neurons or astrocytes were seeded in 96-well plates at a density of 5 × 10^3^ cells per well. At the indicated time points, 10 *μ*L of the CCK-8 solution was added and incubated for 1.5 h. A microplate reader (Tecan, Infinite M200, Austria) was used to measure the absorbance at 450 nm.

### 2.9. Western Blot Analysis

Western blotting was performed as previously described [57]. Brain tissues (cerebral cortex and hippocampus) and cultured cells were extracted in the RIPA lysis buffer with 1% PMSF solution for 30-40 min on ice. After centrifugation (4°C, 12,000 rpm, and 20 min), the supernatant concentration was determined by Coomassie Brilliant Blue G250. The protein samples were denatured and separated on SDS-PAGE gels and then transferred to PVDF membranes (Millipore) on ice. The membranes were blocked in TBST containing 5% nonfat milk and then incubated with the primary antibodies overnight at 4°C, followed by Alexa Fluor-conjugated secondary antibodies at room temperature (RT), using the Odyssey Infrared Imaging System (LI-COR) to detect the signals. ImageJ (National Institutes of Health (NIH)) was used to analyze the intensity of the western blot signals quantitatively, and levels of proteins were expressed by the ratio of protein/*β*-actin or GAPDH (in total protein) or Na, K-ATPase (for membrane extracts) and averaged from three independent experiments.

### 2.10. Semiquantitative Reverse Transcription-PCR

Total RNA isolation of cultured primary astrocytes was performed using the RNAiso Plus (Takara, D9108A). cDNA was synthesized with Maxima Reverse Transcriptase (Takara, Inc., Osaka, Japan). GLT-1 was detected with specific primer pairs (forward primer: 5′-CCT CAT GAG GAT GCT GAA GA-3′, reverse primer: 5′-TCC AGG AAG GCA TCC AGG CTG-3′). *β*-Actin from each sample was also amplified to serve as an internal control. The primer pairs of *β*-actin were 5′-CAC GAT GGA GGG GCC GGA CTC ATC-3′ (forward primer) and 5′-TAA AGA CCT CTA TGC CAA CAC AGT-3′ (reverse primer). The amplified products were electrophoresed on a 1.5% agarose gel stained with GoldView dye and observed using the Quantity One software (Bio-Rad, Hercules, CA, USA). The mRNA levels of genes were normalized to those of *β*-actin and presented as relative to the control.

### 2.11. Immunocytochemistry

Cells were fixed with phosphate-buffered saline (PBS) containing 4% of paraformaldehyde (PFA, *w*/*v*) for 15 min and then were washed three times with PBS. Cells were membrane-permeabilized with 0.2% Triton X-100 in PBS for 20 min. After blocked by 5% bovine serum albumin (BSA) in PBS for 1 h at RT, cells were incubated with primary antibodies overnight at 4°C. Primary antibodies were monoclonal mouse anti-GFAP (1 : 400; Cell Signaling Technology, 3670), polyclonal rabbit anti-GLT-1 antibody (1 : 100; ProteinTech Group, 22515-1-AP), and monoclonal mouse anti-NF-*κ*B p65 (1 : 400; Cell Signaling Technology, 6956). For surface GluA1 immunolabeling, cells were incubated with the primary antibody (anti-AMPA receptor antibody, 1 : 200, Abcam, 183797) without permeabilization after fixation. After washing, the cells were incubated with secondary antibodies (Alexa 488/555-conjugated goat anti-mouse/rabbit anti-IgG (1 : 400, Invitrogen)) for 3-4 h at RT. The cultures were washed three times with PBS and then stained with DAPI for 15 min. The cells were sealed with antifluorescence quenching slides after three washes with PBS. The fluorescent images were acquired by LSM 880 confocal microscopy (Carl Zeiss Corp., Oberkochen, Germany).

### 2.12. Immunofluorescence Analysis

The brains were quickly removed and fixed overnight in PBS containing 4% of PFA and then cryoprotected with 15% and 30% sucrose in PBS for 1 day each until the brain tissues sank to the bottom of the test tube. Then, the brains were embedded in the optimal cutting temperature (OCT) compound and sectioned into 10 *μ*m thick slices until the intact hippocampal structure was observed in the slices through microscopy examination. The sections were mounted on poly-L-lysine-coated slides (Sigma-Aldrich). After fixed with 4% PFA, the slices were permeabilized with 0.5% Triton X-100 (*v*/*v*) in PBS and blocked with 5% of BSA for 1 h at RT and then incubated in primary antibodies at 4°C overnight. Primary antibodies used were as follows: monoclonal mouse anti-GFAP (1 : 200; CST, 3670), polyclonal rabbit anti-GLT-1 antibody (1 : 50; ProteinTech Group, 22515-1-AP), polyclonal mouse anti-MAP2 (1 : 100; ProteinTech Group, 17490-1-AP), polyclonal rabbit anti-NeuN (1 : 100; ProteinTech Group, 26975-1-AP), and polyclonal rabbit anti-AMPA receptor antibody (1 : 200; Abcam, 183797). After washing three times in PBS, the slices were incubated with secondary antibodies (Alexa 488/555-conjugated goat anti-mouse/rabbit anti-IgG) at RT in the dark for 3-4 h. Finally, the slices were incubated with DAPI for 15 min at RT, followed by washing three times with PBS. The slices were sealed with antifluorescence quenching slides and were viewed with an LSM 880 confocal microscopy (Carl Zeiss Corp., Oberkochen, Germany).

### 2.13. Statistical Analysis

All data presented are expressed as arithmetic mean ± standard error of the mean (SEM). All statistical analyses were performed using GraphPad Prism version 8.0. Significant differences were analyzed by the two-sided unpaired Student's *t*-test for two-group comparisons and one-way ANOVA followed by Tukey's *post hoc* test for multiple comparisons. Statistical significance was set at *p* < 0.05.

## 3. Results

### 3.1. Antidepressant Effects of PBMT in CUMS- and CORT-Induced Mouse Models of Depression

**Figure 1:**
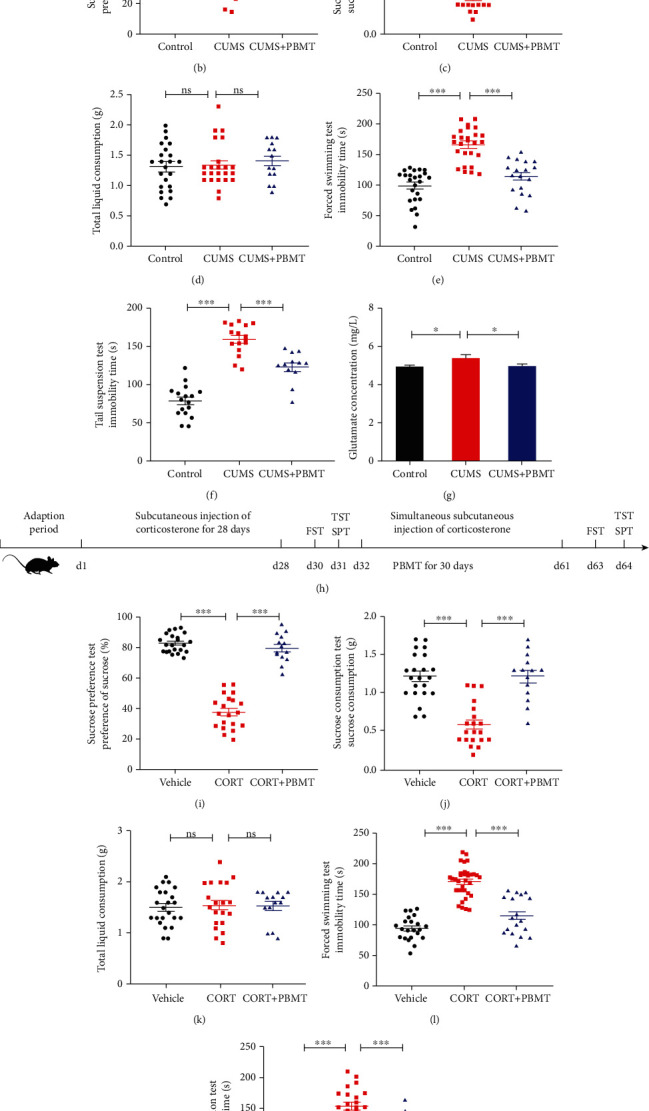
Antidepressant effects of PBMT in CUMS- and CORT-induced mouse models of depression. (a) The experimental design of CUMS, treatment schedule, and behavioral tests. (b–d) PBMT significantly attenuated the decreased sucrose preference induced by CUMS but did not affect total fluid consumption. (e, f) PBMT significantly attenuated the increased immobility time of FST and TST induced by CUMS. (g) Glutamate concentration in CUMS-exposed mice was significantly higher than that in control mice, and PBMT could restore glutamate levels to normal (*n* = 6 per group). (h) The experimental design of CORT, treatment schedule, and behavioral tests. (i–k) PBMT significantly attenuated the decreased sucrose preference induced by CORT but did not affect total fluid consumption. (l, m) PBMT significantly attenuated the increased immobility time of FST and TST induced by CORT. Number of mice used in behavior tests: control/vehicle, *n* = 23 mice; CUMS, *n* = 23 mice; CUMS+PBMT, *n* = 14 mice; CORT, *n* = 20 mice; and CORT+PBMT, *n* = 14 mice. Data represent mean ± SEM. ns: not significant. ^∗^*p* < 0.05, ^∗∗^*p* < 0.01, and ^∗∗∗^*p* < 0.001, one-way ANOVA with Tukey's *post hoc* analysis. CUMS: chronic unpredictable mild stress; CORT: mice treated with corticosterone at a dose of 20 mg/kg for 28 days; PBMT: photobiomodulation therapy; SPT: sucrose preference test; FST: forced swimming test; TST: tail suspension test.

In this study, CUMS and CORT models, which are the widely used animal models of depression, were employed to evaluate the protective effects of PBMT on depressive disorder by the behavior tests, including SPT, FST, and TST. The specific experimental design, stimulation process, and PBMT treatment are depicted in Figures 1(a) and 1(h). Results showed that compared with the control group, mice treated with CUMS and CORT exhibited less preference to sucrose in SPT and SCT trails (Figures 1(b) and 1(c) and Figures 1(i) and 1(j)), without affecting the total liquid consumption (Figures 1(d) and 1(k)). Furthermore, CUMS and CORT mice showed a significant increase of immobility time in both FST (Figures 1(e) and 1(l)) and TST (Figures 1(f) and 1(m)). In contrast, these depressive-like behaviors and phenotypes were significantly reversed after treated with PBMT at a dose of 2 J/cm^2^ for 10 min/day for 30 days.

Generally, animal behaviors are closely connected with the levels of neurotransmitters in the brain. Accumulating studies suggest that the glutamatergic system dysfunction plays a vital role in the pathophysiology and treatment of depression [64–66]. Glutamate levels are elevated in the cerebrospinal fluid, plasma, serum and brains in MDD patients [65, 67]. Thus, we detected the levels of glutamate in the cerebral cortex and hippocampus of mice. As indicated in Figure 1(g) and Figure S1, CUMS treatment significantly increased glutamate levels, which were reversed by PBMT. Collectively, PBMT showed potential antidepressive effects in mice treated with CUMS or CORT. These findings together with the observations from behavioral tests indicated the antidepressive effect of PBMT.

### 3.2. Effects of PBMT on Loss of Astrocytes and Expression of GLT-1 in the Cortex and Hippocampus of Depressed Mice

**Figure 2:**
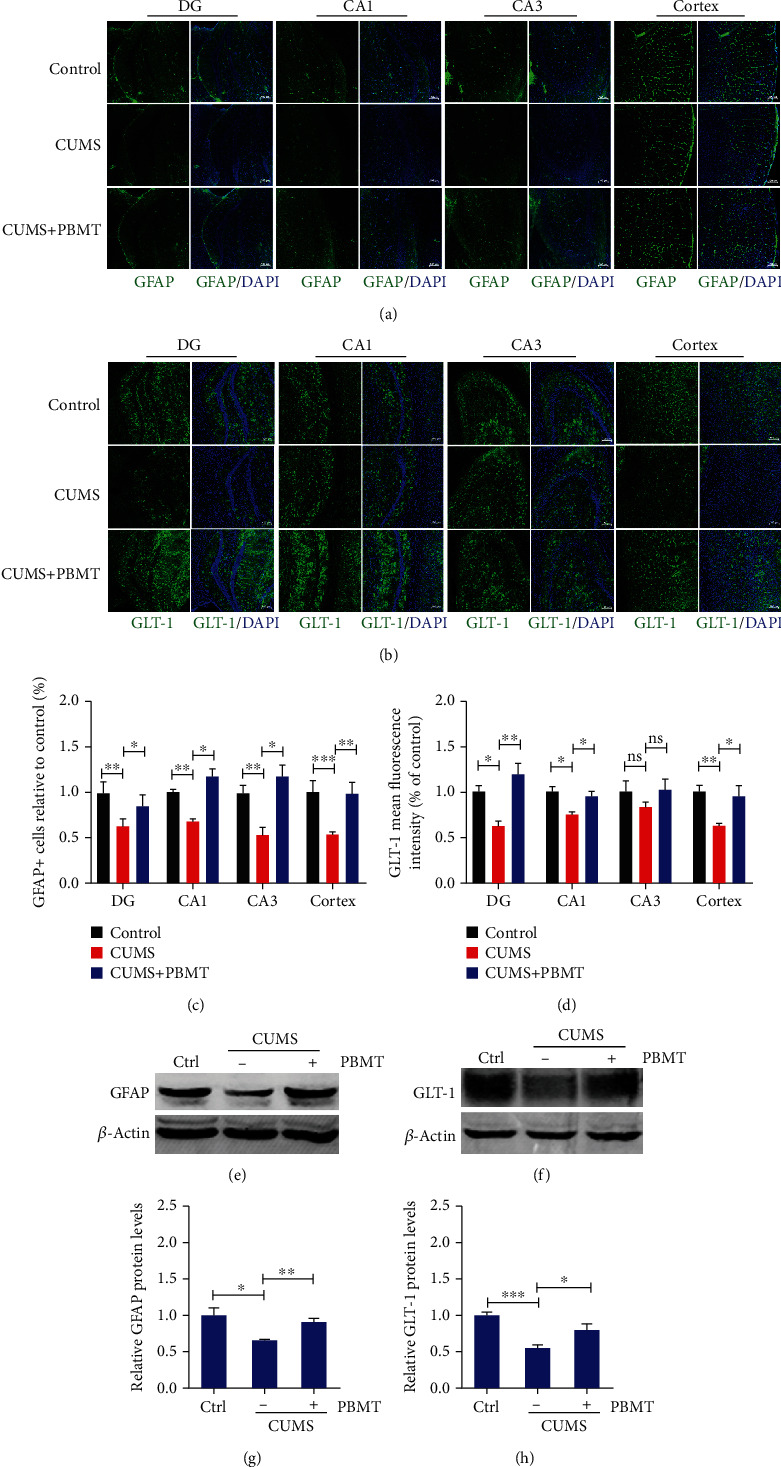
Effects of PBMT on the loss of astrocytes and expression of GLT-1 in the cortex and hippocampus of depressed mice. (a) Representative immunofluorescent images of GFAP in the hippocampal and cortex regions of each group. Nuclei were counterstained with DAPI (blue). Scale bar: 100 *μ*m (*n* = 6 per group). (b) Representative immunofluorescent images of GLT-1 in the hippocampal and cortex regions of each group. Scale bar: 100 *μ*m (*n* = 6 per group). (c) Quantification analysis of GFAP-positive cells in the hippocampal and cortex regions of different groups. (d) Quantification analysis of GLT-1 in the hippocampal and cortex regions of different groups. (e–h) GFAP and GLT-1 expression detected by western blot in the cortex and hippocampus lysates from mice exposed to CUMS with or without PBMT (*n* = 9 mice for each group). Data represent mean ± SEM. ns: not significant. ^∗^*p* < 0.05, ^∗∗^*p* < 0.01, and ^∗∗∗^*p* < 0.001, one-way ANOVA with Tukey's *post hoc* analysis. DAPI: 4′,6-diamidino-2-phenylindole; GFAP: glial fibrillary acidic protein; GLT-1: glutamate transporter-1.

Astrocytes are crucial for glutamate uptake and metabolism in the central nervous system [12]. As the result shown, immunostaining revealed that GFAP-positive cells are markedly decreased in the cortex and three regions (DG, CA1, and CA3) of the hippocampus after CUMS treatment (Figures 2(a) and 2(c)), and this inhibition was reversed by the treatment of PBMT. Additionally, western blot analysis of GFAP was consistent with that result of immunostaining (Figures 2(e) and 2(g)). GLT-1 is mostly expressed on astrocytes and responsible for the reuptake of over 90% of glutamate [13]. We thus detected the role of GLT-1 in the antidepressant effects of PBMT by western blot and immunostaining analysis in the cortex and hippocampus. As indicated in Figures 2(b), 2(d), 2(f), and 2(h), PBMT significantly attenuated the CUMS-induced reduction of GLT-1 in these regions. Furthermore, we found that chronic injections of CORT decreased GFAP-positive cells in the cortex region, and the protein level of GLT-1 was also decreased, whereas both of which were reversed by PBMT (Figure S2a-2d). Overall, these results suggested that PBMT was likely to reduce glutamate levels by upregulating GLT-1 expression to exert neuroprotective effects.

### 3.3. Effects of PBMT on Dendritic Atrophy and Expression of GluA1 in the Cortex and Hippocampus of Depressed Mice

**Figure 3:**
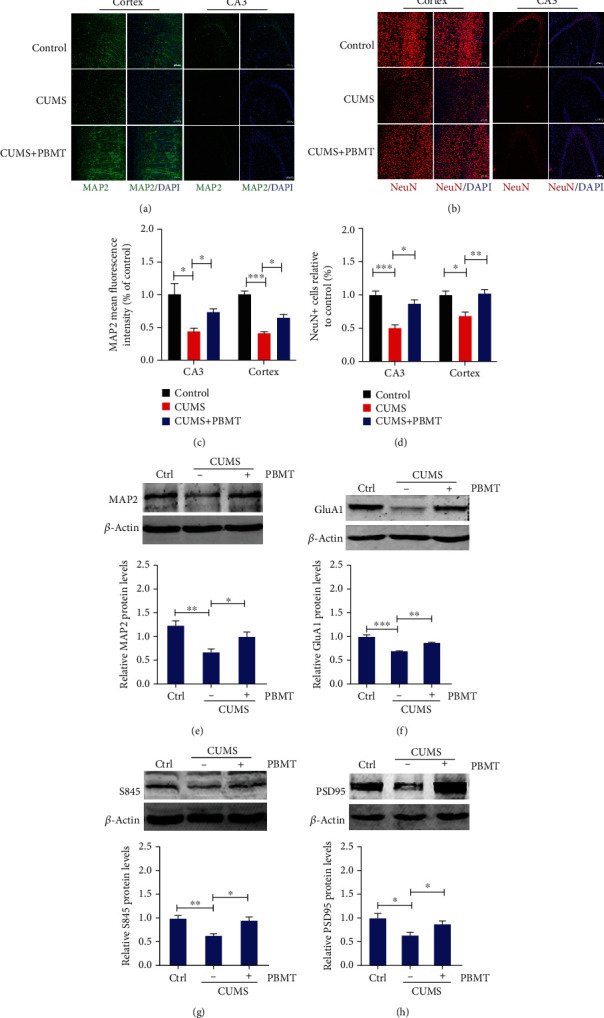
Effects of PBMT on dendritic atrophy and expression of GluA1 in the cortex and hippocampus of depressed mice. (a) Typical staining of MAP2 (green) in the cortex and hippocampal regions from CUMS mice with or without PBMT and the control group. Nuclei were counterstained with DAPI (blue). Scale bar, 100 *μ*m (*n* = 6 per group). (b) Representative immunofluorescent image analysis of NeuN-positive cells (red) in the hippocampal and cortex regions of each group; scale bar represents 100 *μ*m. (c) Quantification of MAP2 density in the hippocampal and cortex regions of different groups. (d) Quantitative analyses of the NeuN-positive cells in the hippocampal and cortex regions of different groups. (e) Western blot and quantification analysis of MAP2 from control vs. chronic stressed mice without or with PBMT treatment (*n* = 6-8 per group). (f) Western blot and quantification analysis of GluA1 from control vs. chronic stressed mice without or with PBMT treatment. (g) Representative western blot and quantification analysis of S845 in chronic stressed mice under the treatment with or without PBMT. (h) Western blot and quantification analysis of PSD95 from control vs. chronic stressed mice without or with PBMT treatment. All the data represent mean ± SEM. ^∗^*p* < 0.05, ^∗∗^*p* < 0.01, and ^∗∗∗^*p* < 0.001, one-way ANOVA with Tukey's *post hoc* analysis. MAP2: microtubule-associated protein 2; PSD95: postsynaptic density 95.

Because of the physical proximity of glial processes and neurons and the dynamic interactions between these two cell types, any changes in the number or morphology of glial cells will also influence neuronal function [68]. To further investigate the effects of PBMT on CUMS-induced dendrite damage, brain sections were stained with MAP2, which plays an important role in the growth, differentiation, and plasticity of neurons [69]. Compared to the control group, CUMS mice had less MAP2 fluorescent staining intensity in both the cortex and hippocampal CA3 region (Figures 3(a) and 3(c)), and the decrease in the numbers of dendrites was shown in immunostaining images, indicating that the dendrites in CUMS-treated mice were likely atrophied, which were consistent with western blot analysis of MAP2 (Figure 3(e)). In contrast, dendritic atrophy was improved in PBMT-treated CUMS mice. Furthermore, we also analyzed the effect of PBMT on the number of neurons; in contrast to the CUMS group, PBMT treatment significantly increased the number of NeuN-positive cells in the cortex and hippocampal CA3 region (Figures 3(b) and 3(d)). Increased stimulation of AMPA receptors leads to an increased influx of calcium and sodium and a strengthening of intracellular signaling, contributing to improved neuroplasticity and neuronal function [70, 71]. GluA1 is an important subunit of AMPA receptors, and serine at position 845 is a major phosphorylation site [32]. Our results suggested that chronic stress decreased the expression of GluA1 and phosphorylation levels of S845, whereas all these changes were reversed by PBMT (Figures 3(f) and 3(g)). Consistent with reduced expression of GluA1, we observed significant decreases in expression of the postsynaptic protein PSD95 compared with controls. Similarly, PBMT reversed this change (Figure 3(h)). In addition, we found that chronic injections of CORT decreased MAP2 mean fluorescence intensity in the cortex region, and protein levels of MAP2 and GluA1 were also decreased, whereas all of which were reversed by PBMT (Figure S2e-2j). Taken together, these results suggested that PBMT alleviated dendritic atrophy and upregulated the expression of AMPA receptors in mice of depression.

### 3.4. PBMT Upregulates GLT-1 Expression by Activating the PI3K/Akt/NF-*κ*B Signaling Pathway in CORT-Treated Primary Astrocytes

**Figure 4:**
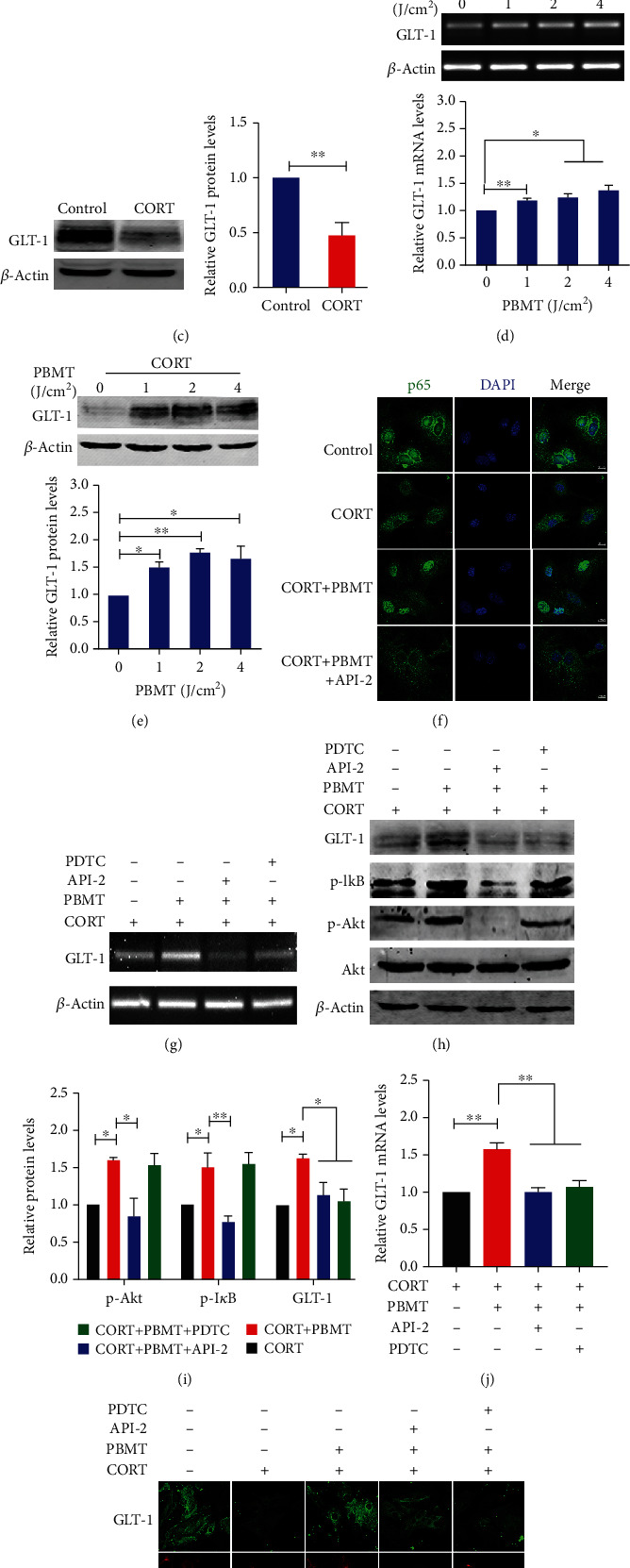
PBMT upregulates GLT-1 expression by activating the PI3K/Akt/NF-*κ*B signaling pathway in CORT-treated primary astrocytes. (a) CORT induced reduced viability of primary cultured astrocytes in dose-dependent manners as measured using the CCK-8 assay. The ordinate represents the percentage of cell survival compared with controls as measured by the CCK-8 assay. (b) Primary astrocytes were exposed to 200 *μΜ* corticosterone followed by irradiation with PBMT at 1 J/cm^2^, 2 J/cm^2^, or 4 J/cm^2^, respectively. Cell viability was assessed by the CCK-8 assay after 24 h. (c) Western blot and quantification analysis of GLT-1 expression in 200 *μΜ* CORT-treated primary astrocytes. (d, e) Representative PCR and western blot and quantification analysis that PBMT increases GLT-1 mRNA and protein levels in a dose-dependent manner. (f) Representative immunofluorescent images of p65 (green) in primary astrocytes under the indicated treatments. Staining with DAPI (blue) to visualize the nucleus. Scale bar: 10 *μ*m. (g, j) GLT-1 mRNA levels were detected by PCR stimulated with CORT and/or PBMT in the preference of API-2 (6 *μΜ*) and PDTC (8 *μΜ*) in primary astrocytes. (h, i) Representative western blot and quantification analysis of GLT-1 stimulated with CORT and/or PBMT in the preference of API-2 (6 *μΜ*) and PDTC (8 *μΜ*) in primary astrocytes. (k) Representative immunofluorescent images of GLT-1 (green) in astrocytes (red) under the indicated treatments. Staining with DAPI (blue) to visualize the nucleus. Scale bar: 20 *μ*m. All the data represent mean ± SEM. ^∗^*p* < 0.05, ^∗∗^*p* < 0.01, and ^∗∗∗^*p* < 0.001. Significant differences were analyzed by the two-sided unpaired Student's *t*-test for two-group comparisons and one-way ANOVA followed by Tukey's *post hoc* test for multiple comparisons. CORT: primary astrocytes treated with corticosterone.

CORT, a major glucocorticoid secreted by the HPA axis in rodents, has been found as one of the detrimental factors for chronic stress-evoked neurotoxic effects [72]. To further investigate the mechanism of PBMT *in vitro*, primary astrocytes were treated with CORT to establish a cell stress model in the brain of depression model mice. To determine the appropriate concentration of CORT, primary astrocytes were exposed to an increasing concentration of CORT (25-400 *μΜ*) for 24 h. As shown in Figure 4(a), treatment with 200 *μΜ* CORT for 24 h significantly decreased astrocytes viability to 59.45%. To further examine the optimal dose of PBMT, primary astrocytes were exposed to 200 *μΜ* CORT followed by exposure of cells to different doses of PBMT (1, 2, or 4 J/cm^2^). The viability of cells was measured after 24 h by using the CCK-8 assay. As shown in Figure 4(b), cell viability was evidently increased by PBMT in a dose-dependent manner. The cell viability was significantly increased at the dose of 2 and 4 J/cm^2^, and the dose of 2 J/cm^2^ was selected as the optimum irradiation dose in our following studies to minimize the thermal effect.

We next investigated the effect of CORT on the expression of GLT-1 in astrocytes; western blot analysis showed that the levels of GLT-1 protein were decreased in CORT-treated primary astrocytes (Figure 4(c)). We then examined if PBMT could regulate GLT-1 expression; our results showed that protein and mRNA levels of GLT-1 were increased in a dose-dependent manner, with a statistically significant increase observed at 2 J/cm^2^ PBMT (Figures 4(d) and 4(e)).

It is reported that NF-*κ*B is a transcription factor of GLT-1, and previous studies have shown that activation of the PI3K/Akt/NF-*κ*B signaling pathway plays a key role in upregulating GLT-1 expression [73–75]. The GLT-1 promoter contains three NF-*κ*B consensus sites, and previous studies found that EGF and TGF-*α* enhance the expression of GLT-1 through NF-*κ*B signaling [76]. Thus, we speculated that PBMT could upregulate the expression of GLT-1 through activating NF-*κ*B even in CORT-treated primary astrocytes. As shown in Figure 4(f), p65 levels in the nucleus increased after PBMT. However, the translocation of p65 into the nucleus was obviously inhibited after the cells were preincubated with API-2 (specific inhibitor for Akt). We next tested the specific inhibitors of these pathways in western blot assays: API-2 and PDTC (for Akt and NF-*κ*B, respectively). We demonstrated that PBMT increased the expression of GLT-1, which was reversed by API-2 and PDTC, respectively, suggesting that the Akt/NF-*κ*B signaling pathway is obligatory for PBMT-stimulated GLT-1 upregulation even in CORT-treated astrocytes (Figures 4(g)–4(j)). As shown in Figures 4(h) and 4(i), GLT-1, p-Akt, and p-I*κ*B levels were increased after PBMT treatment. API-2 reduced the levels of p-Akt and p-I*κ*B induced by PBMT and also blocked the upregulation of PBMT to GLT-1 in protein and mRNA levels. These results were also confirmed by immunofluorescent staining with antibodies against GLT-1 in primary astrocytes (Figure 4(k)). Taken together, these results revealed that PBMT activated the Akt/NF-*κ*B signaling pathway to increase the expression of GLT-1.

### 3.5. PBMT Promotes AMPA Receptor Insertion through Activation of PKA in CORT-Treated Primary Neurons

**Figure 5:**
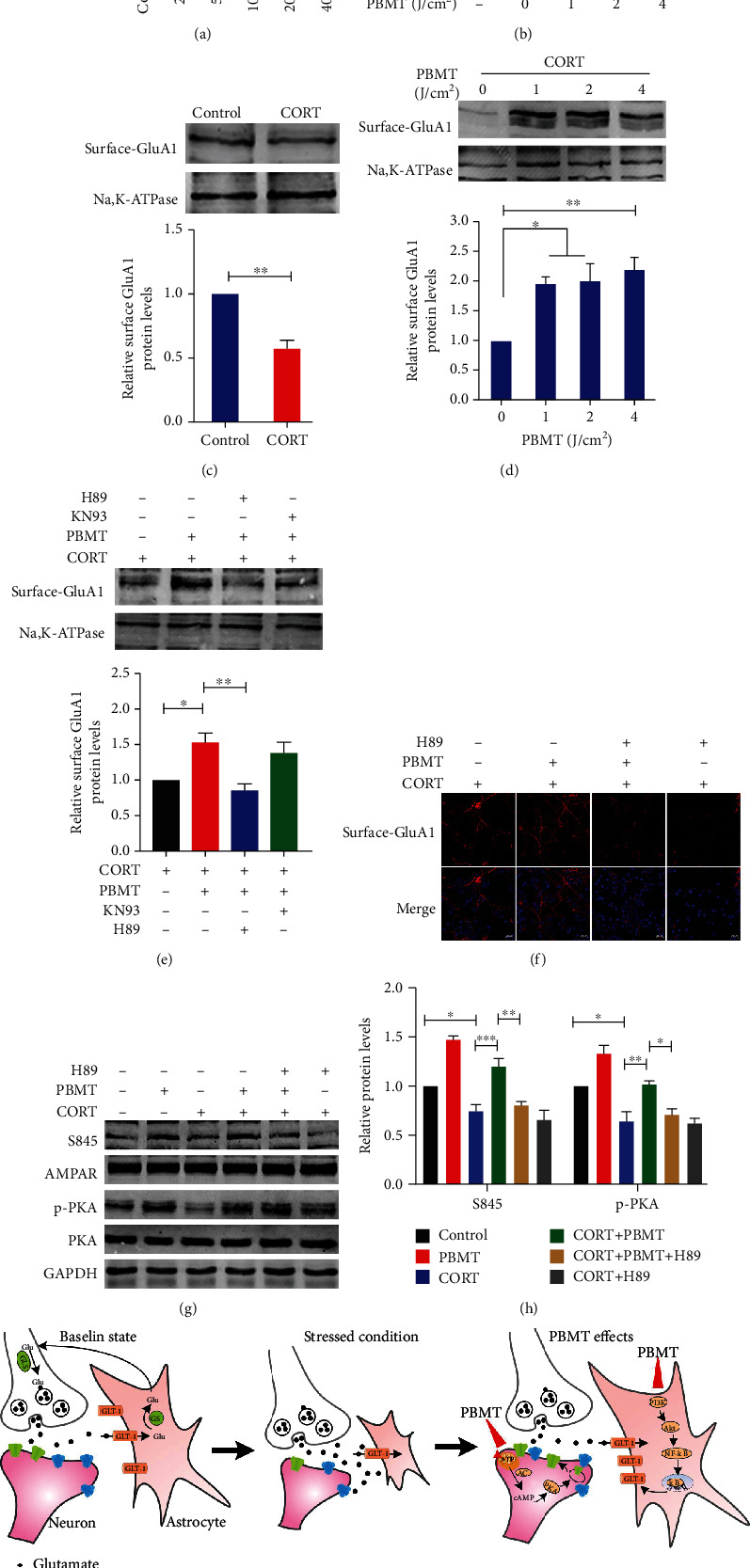
PBMT promotes AMPA receptor insertion through activation of PKA in CORT-treated primary neurons. (a) CORT induced reduced viability of primary cultured neurons in dose-dependent manners as measured using the CCK-8 assay. The ordinate represents the percentage of cell survival compared with controls. (b) Primary neurons were exposed to 100 *μΜ* CORT followed by irradiation with PBMT at 1 J/cm^2^, 2 J/cm^2^, or 4 J/cm^2^, respectively. Cell viability was assessed by the CCK-8 assay after 24 h. (c) Representative western blot and quantification analysis of surface levels of AMPA receptor subunit GluA1 in 100 *μΜ* CORT-treated primary neurons. (d) Representative western blot and quantification analysis of the dose-dependent effect of PBMT on surface levels of AMPA receptor subunit GluA1 expression after 24 h. (e) Representative western blot and quantification analysis of surface GluA1 stimulated with CORT and/or PBMT in the preference of H89 (20 *μΜ*) and KN93 (10 *μΜ*) in primary neurons. (f) Representative immunofluorescent images of surface GluA1 (red) in neurons under the indicated treatments. Staining with DAPI (blue) to visualize the nucleus. Scale bar: 20 *μ*m. (g, h) Representative western blot and quantification analysis of S845, PKA, and p-PKA stimulated with CORT and/or PBMT in the preference of H89 (20 *μΜ*) and KN93 (10 *μΜ*) in primary neurons. (i) Schematic representation of the signaling pathway for PBMT ameliorates glutamatergic dysfunction. All the data represent mean ± SEM. ^∗^*p* < 0.05, ^∗∗^*p* < 0.01, and ^∗∗∗^*p* < 0.001. Significant differences were analyzed by the two-sided unpaired Student's *t*-test for two-group comparisons and one-way ANOVA followed by Tukey's *post hoc* test for multiple comparisons. CORT: primary neurons treated with corticosterone.

Repeat previous experiments to determine the optimal concentration of CORT-injured neurons. We exposed primary neurons to an increasing concentration of CORT (25-200 *μΜ*) for 24 h. As shown in Figure 5(a), after being treated with 100 *μ*M CORT for 24 h, the viability of primary neurons decreased to 61.15%. It was also observed that PBMT significantly increased cell viability in a dose-dependent manner (Figure 5(b)).

AMPA receptors mediate the vast majority of excitatory neurotransmission in the brain [28]. Previous studies have shown that chronic stress selectively decreases GluA1 AMPA receptor subunits levels and the function of specific synapses. The data in Figure 5(c) has shown that 100 *μΜ* CORT treatment significantly reduced the expression of surface GluA1 subunits; however, even in CORT-treated neurons, PBMT increased the level of GluA1 on the membrane (Figure 5(d)).

It is well known that protein kinase A (PKA) and Ca2^+^/calmodulin-dependent protein kinase II (CaMKII) are related to the trafficking of AMPA receptors [77]. To determine which kinase plays a key role in upregulating AMPA receptor levels on membranes, we tested the level of GluA1 on the membrane after adding PKA and CaMKII inhibitors H89 and KN93, respectively. Western blot analysis suggested that PBMT upregulated surface AMPA receptor expression in CORT-treated neurons, which was reversed by pretreatment with H89, but not KN93 (Figure 5(e)). These results were also confirmed by immunofluorescent staining with the anti-AMPA receptor antibody in primary neurons (Figure 5(f)). Studies have shown that PKA phosphorylates the GluA1 at serine 845 [78]. So, we next examined the effect of PBMT on phosphorylation levels of S845 and PKA. As shown in Figures 5(g) and 5(h), H89 reduced the level of p-PKA and phosphorylation level at S845 sites of GluA1 induced by PBMT. This result further suggests that PBMT phosphorylates the S845 site which requires activation of PKA.

## 4. Discussion

Abundant pieces of evidence have indicated the pivotal role of stress in the occurrence of depression disorder, in which the dysfunction of the glutamatergic neurotransmission has been implicated [64–66]. Consistent with these previous reports, our study also noticed an obvious increased glutamate level in chronic stress-induced depression. PBMT is a novel and nonthermal irradiation that use red to NIR light, which has shown good effects on the treatment of stroke [79], traumatic brain injury [80], neurodegenerative disease [81], and psychological disorders [82–84]. Previous studies of our group demonstrated that PBMT ameliorated dendritic atrophy via upregulation of BDNF [57] and reduced A*β* levels by activating the PKA/SIRT1 signaling pathway in an Alzheimer's disease model [50]. Therefore, we hypothesized that PBMT also has neuroprotective effects on excitotoxicity induced by glutamate accumulation in the animal model of depression. As our expectation, we found that PBMT treatment obviously decreased the glutamate levels in CUMS-induced depression mice.

The concentrations of extracellular glutamate are tightly regulated by glutamate transporters, especially GLT-1, which is mainly expressed by astrocytes [85]. Clinical postmortem studies of depressed patients and animal models of depression reported the loss of glial cells and lower expression of GLT-1 in the PFC and hippocampus [60, 86–88]. Similarly, we found astrocyte loss and the low expression of GLT-1 in the cerebral cortex and hippocampus after CUMS, which may contribute to the high levels of glutamate. But these changes were reversed by 30 days of PBMT treatment. We found a significant reduction of GFAP^+^ and NeuN^+^ cells following CUMS, which may be related to necrosis and/or apoptosis that need to be further investigated.

Considering the role of astrocytes in nurturing neurons and maintaining synaptic transmission, it is not surprising that postmortem studies and animal models of depression have reported neuronal alterations in the PFC and hippocampus [15, 89–93]. In glutamatergic synapses, the ionotropic AMPA receptor located on the postsynaptic membrane of neurons mediates most of the fast transmission, and changes in its trafficking have been considered to be a core mechanism for synaptic plasticity [94]. However, recent studies have found that chronic stress downregulated the mRNA and protein levels of GluA1 AMPA receptor subunits in the CA1 region of the hippocampus [33, 95], which is consistent with our results. We demonstrated the ability of PBMT to rescue CUMS-induced dendritic atrophy and AMPA receptor downregulation.

In CORT-treated primary neurons, PBMT attenuated the decrease of surface GluA1 protein levels and increased the phosphorylation level of GluA1 at serine 845, which promotes AMPA receptor exocytosis. Protein kinase A (PKA) is necessary for phosphorylation of serine at position 845 of GluA1 [77]. How could PBMT activate the PKA signaling pathway? cAMP, a molecule derived from ATP, is the first step in the activation of PKA, which can then phosphorylate a variety of targets, further controlling the biosynthesis of DNA and RNA. It has been found that the biological effects induced by PBMT are caused by the absorption of photons by intracellular photoreceptors (such as cytochrome c oxidase (CcO)), which leads to the excited state of electrons and the acceleration of electron transfer reaction [49]. More electron transport necessarily leads to increased production of ATP, which further increases the level of cAMP to activate PKA [96, 97]. Our previous study has found that PBMT can accelerate electron transfer by activating CcO, resulting in increased ATP synthesis, which further increases the level of cAMP to activate PKA [50]. It is worth noting that targeting CcO is an important pathway for improving depression, as mitochondrial dysfunction has been observed in brains with depression [98]. Accordingly, we speculate that PBMT can promote the phosphorylation and surface levels of AMPA receptors by activating the cAMP/PKA signaling pathway. In fact, the mechanism of AMPA receptor exocytosis and endocytosis is very complicated, our study only discussed the change of GluA1 subunits of AMPA receptors and other AMPA receptor subunits, and glutamate receptors can also be used as a research object which needs further study.

As summarized in Figure 5(i), this study clarified for the first time that the mechanism of PBMT to improve depressive behaviors is related to the modulation of glutamatergic neurotransmission. Akt/NF-*κ*B and cAMP/PKA pathways were proved to, at least in part, be involved in the action of PBMT on astrocytes and neurons. Our findings revealed some of the underlying mechanisms for PBMT-induced neuroprotective effects against injury and provided further insights into the potential for new therapeutic treatments of depression.

## 5. Conclusions

In summary, the accumulation of glutamate induced by CUMS and its excitotoxicity may be related to the loss of astrocytes and the decreased expression of GLT-1. Because of the dynamic interactions between astrocytes and neurons, any changes in the number or morphology of glial cells will also affect the function of neurons. So, it is not surprising that we found dendrites were atrophied and AMPA receptor expression was decreased in depressed mice. Furthermore, the antidepressant effect of PBMT may be related to the upregulation of GLT-1 expression and enhancement of extrasynaptic glutamate clearance. Although cultured primary neurons, astrocytes, and their treatment with corticosterone may not be completely similar to neurons and astrocytes in the brain of depression patients, our research suggested that upregulating GLT-1 may be a crucial step for reconstruction or improvement of glutamate uptake by PBMT. A better understanding of the regulatory mechanism of PBMT may provide a novel treatment to control the progression of depression.

## Data Availability

Raw data is available from the corresponding author upon reasonable request.
