# Supplementary material for: Photobiomodulation Therapy Ameliorates Glutamatergic Dysfunction in Mice with Chronic Unpredictable Mild Stress-Induced Depression
Source: Oxid Med Cell Longev. 2021 Mar 29;2021:6678276. doi: 10.1155/2021/6678276 (PMC8024102; doi:10.1155/2021/6678276)
Supplement: Supplementary Materials — Additional supporting information may be found online in the Supporting Information section. [file 6678276.f1.docx]

**Supplemental Data**

**Method**

**Liquid Chromatography-Mass Spectrometry for Glutamate Quantification**

Brain tissues (cerebral cortex and hippocampus) were weighed, methanol solution and several glass grinding beads were added to grind for 5 min. Then vibrated on a vortex instrument for 10 min, and then kept at 4℃ for 20 min. After centrifugation (12000 rpm, 4℃, 10 min), the supernatant was subsequently analyzed using liquid chromatography-mass spectrometry (LC-MS) to determine glutamate levels. The instrument used in this experiment is Shimadzu Ultra High-Performance Liquid Chromatograph (LC-30AT) connected to SCIEX 5600+ mass spectrometer. The specific parameters are as follows: Component Name: Hybrid Quadrupole-TOF LC/MS/MS Mass Spectrometer, Component ID: TripleTOF 5600+, Manufacturer: AB Sciex, Instruments; Shimadzu LC30AD. The chromatographic column is ACQUITY UPLC BEH HILIC Column (1.7 µm, 2.1 mm×100 mm), the mobile phase is A: acetonitrile, B: 0.1% formic acid aqueous solution, gradient elution.

**Supplemental Figure**

**
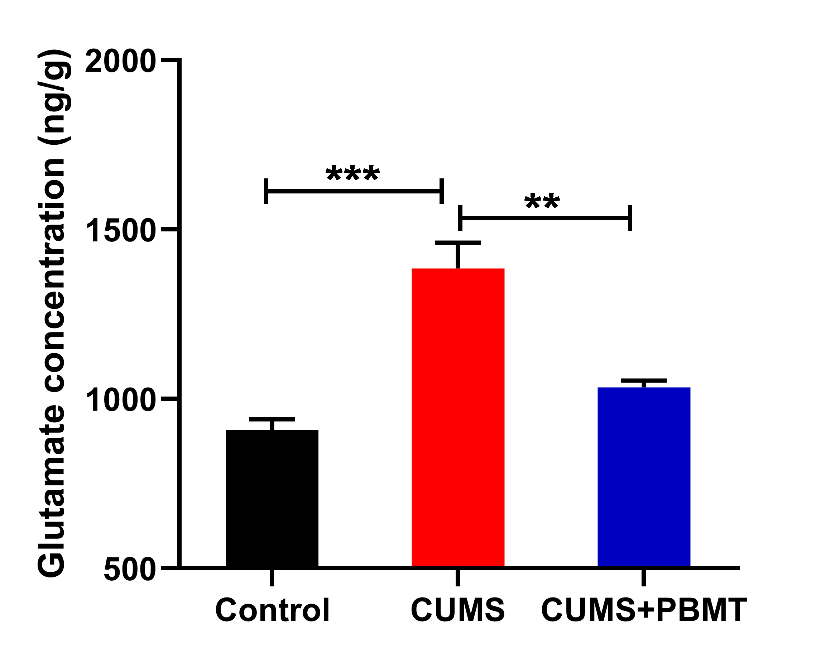
**

**Figure S1.** Extracellular glutamate in CUMS-exposed mice was significantly higher than that in control mice, and PBMT could restore glutamate levels to normal (*n* = 6 per group). Data represent mean ± SEM; **p* < 0.05, ***p* < 0.01, ****p* < 0.001, one-way ANOVA with Tukey’s *post hoc* analysis.

**
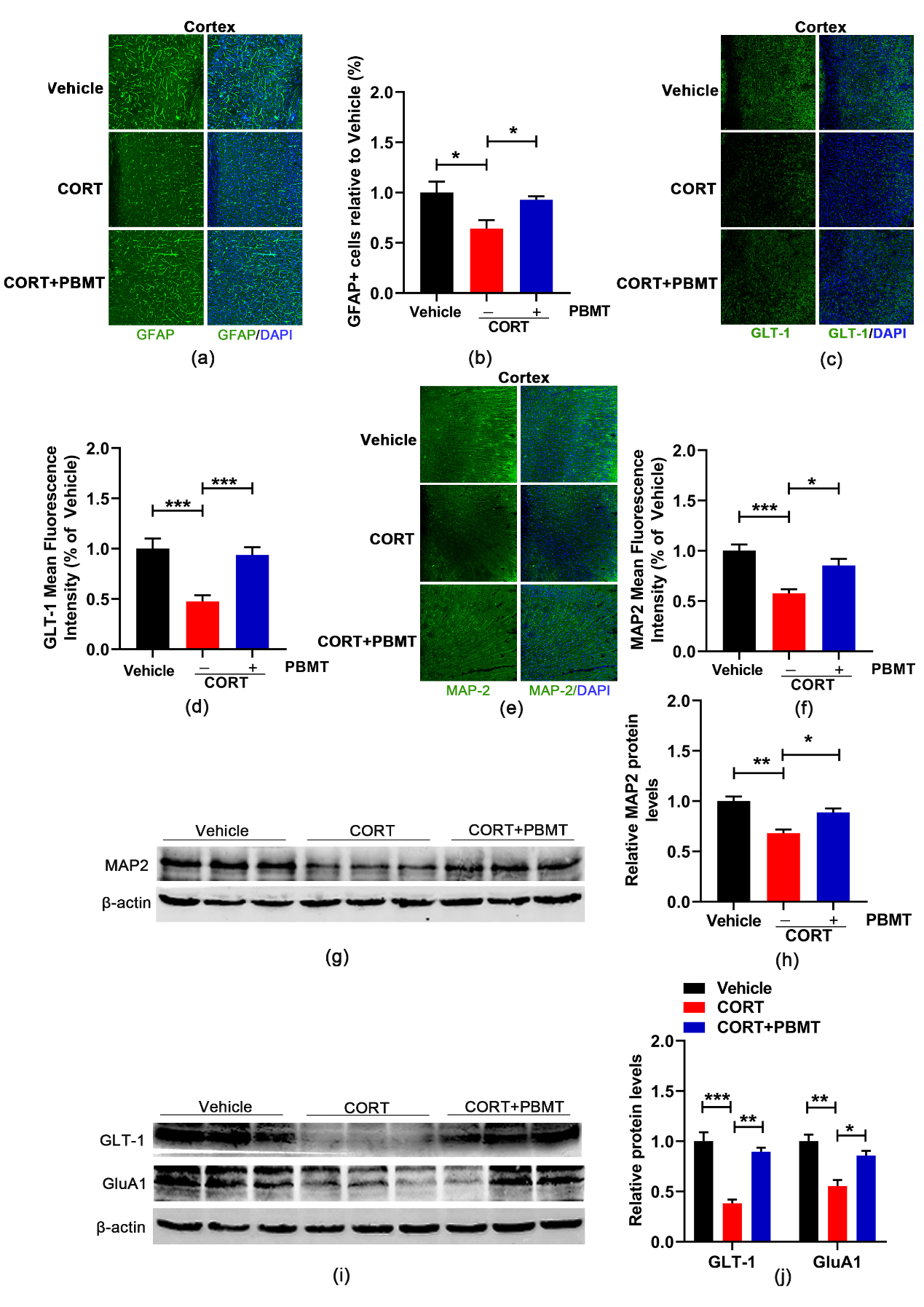
**

**Figure S2. Effects of PBMT on Loss of Astrocytes, Expression of GLT-1, Dendritic Atrophy and Expression of GluA1 in the Cortex of** **CORT-Injected Mice**

(a) Representative immunofluorescent images of GFAP in cortex region of each group. Nuclei were counterstained with DAPI (blue). Scale bar: 100 μm (*n* =6 per group). (b) Quantification analysis of GFAP-positive cell in hippocampal and cortex regions of different groups. (c) Representative immunofluorescent images of GLT-1 in hippocampus and cortex regions of each group. Scale bar: 100 μm (*n* = 6 per group). (d) Quantification analysis of GLT-1 in cortex regions of different groups. (e) Typical staining of MAP2 (green) in cortex region from CUMS mice with or without PBMT and control group. Nuclei were counterstained with DAPI (blue). Scale bar, 100 μm. (*n* = 4-5 per group). (f) Quantification of MAP2 mean fluorescence intensity in cortex region of different groups. (g-h) Western blot and quantification analysis of MAP2 from Vehicle vs. chronic CORT-injected mice without or with PBMT treatment (*n* = 6-8 per group). (i-j) Western blot and quantification analysis of GLT-1 and GluA1 from Vehicle vs. chronic CORT-injected mice without or with PBMT treatment (*n* = 6-8 per group). Data represent mean ± SEM; **p* < 0.05, ***p* < 0.01, ****p* < 0.001, one-way ANOVA with Tukey’s *post hoc* analysis. GFAP, glial fibrillary acidic protein; GLT-1, glutamate transporter 1; MAP2: microtubule-associated protein 2; CORT: mice treated with corticosterone at a dose of 20 mg/kg for 28 days.

**Supplemental Tables**

**Table S1.** Laser parameters used on the mice

| Light source | Semiconductor laser |
| --- | --- |
| Wavelength (nm) | 635 |
| Emission | Continuous wave |
| Distance to the mice (cm) | 12 ± 1 |
| Spot size (cm^2^) | 7.06 |
| Power density over the mice head (mW/cm^2^) | 10.05 |
| Power density in the mice hippocampus (mW/cm^2^) | 3.015 |
| Irradiation time (min) | 10 |
| Energy density in the mice hippocampus (J/cm^2^) | 2 |
| Frequency of treatment sessions | Once a day |
| Number of treatment sessions | 30 sessions |
| The transmittance of the laser through the skin and skull to the interior of hippocampus was measured to be about 30%. | |

**Table S2**. Laser parameters used on the cells

| Light source | Semiconductor laser |
| --- | --- |
| Wavelength (nm) | 635 |
| Emission | Continuous wave |
| Distance to the cells (cm) | 20 ± 1 |
| Spot size (cm^2^) | 7.06 |
| Power density over the cells (mW/cm^2^) | 3.3 |
| Irradiation time (min) | 1.25/2.5/5 |
| Energy density (J/cm^2^) | 1/2/4 |
